# Full etiologic spectrum of pediatric severe to profound hearing loss of consecutive 119 cases

**DOI:** 10.1038/s41598-022-16421-x

**Published:** 2022-07-19

**Authors:** Young Seok Kim, Yoonjoong Kim, Hyoung Won Jeon, Nayoung Yi, Sang-Yeon Lee, Yehree Kim, Jin Hee Han, Min Young Kim, Bo Hye Kim, Hyeong Yun Choi, Marge Carandang, Ja-Won Koo, Bong Jik Kim, Yun Jung Bae, Byung Yoon Choi

**Affiliations:** 1 Department of Otorhinolaryngology-Head and Neck Surgery, Seoul National University Hospital, Seoul, South Korea; 2 Department of Otorhinolaryngology-Head and Neck Surgery, Seoul National University Bundang Hospital, 300 Gumi-dong, Bundang-gu, Seongnam-si, Kyunggi-do 463-707 South Korea; 3 Department of Otolaryngology-Head and Neck Surgery, Chungnam National University Sejong Hospital, Sejong, South Korea; 4 College of Medicine, Chungnam National University, Daejeon, South Korea; 5 College of Medicine, Seoul National University, Seoul, South Korea; 6 Information Science Major, University of Maryland, College Park, MD USA; 7 Department of Otorhinolaryngology-Head and Neck Surgery, East Avenue Medical Center, Metro Manila, Philippines; 8 Department of Radiology, Seoul National University Bundang Hospital, Seongnam, South Korea

**Keywords:** Genetics, Medical research

## Abstract

Determining the etiology of severe-to-profound sensorineural hearing loss (SP-SNHL) in pediatric subjects is particularly important in aiding the decision for auditory rehabilitation. We aimed to update the etiologic spectrum of pediatric SP-SNHL by combining internal auditory canal (IAC)-MRI with comprehensive and state-of-the-art genetic testings. From May 2013 to September 2020, 119 cochlear implantees under the age of 15 years with SP-SNHL were all prospectively recruited. They were subjected to genetic tests, including exome sequencing, and IAC-MRI for etiologic diagnosis. Strict interpretation of results were made based on ACMG/AMP guidelines and by an experienced neuroradiologist. The etiology was determined in of 65.5% (78/119) of our cohort. If only one of the two tests was done, the etiologic diagnostic rate would be reduced by at least 21.8%. Notably, cochlear nerve deficiency (n = 20) detected by IAC-MRI topped the etiology list of our cohort, followed by DFNB4 (n = 18), DFNB1 (n = 10), DFNB9 (n = 10) and periventricular leukomalacia associated with congenital CMV infection (n = 8). Simultaneous application of state-of-the-art genetic tests and IAC-MRI is essential for etiologic diagnosis, and if lesions of the auditory nerve or central nerve system are carefully examined on an MRI, we can identify the cause of deafness in more than 65% of pediatric SP-SNHL cases.

## Introduction

Even though pediatric patients without etiologic confirmation could receive the proper auditory rehabilitation based on the hearing status, identifying the etiology of severe-to-profound sensorineural hearing loss (SP-SNHL) puts us obviously in a better position to predict the natural course of the hearing loss and outcome of each hearing rehabilitation in pediatric patients, leading to the most appropriate auditory rehabilitation. Awareness of the etiology of SP-SNHL sometimes, helps to make a decision of timely cochlear implantation (CI). In some cases, parents hesitate before deciding on CI for their child, especially when the cause of SP-SNHL remains unknown. This could result in prolonged auditory deprivation and interference with language development^1^. Knowing the etiology—either molecular genetically or radiologically—will help convince parents and prevent unnecessary delay of hearing rehabilitation. For example, *SLC26A4* and *GJB2* variants are the major cause of SP-SNHL with different progressive features from each other, so when they are confirmed, hearing rehabilitation can be planned accordingly^2,3^. And it has been reported that subjects with *OTOF*-related auditory neuropathy spectrum disorder could be managed effectively with timely CI after molecular etiologic confirmation, which would have been difficult without genetic confirmation^4,5^. In some cases etiologic diagnosis of hearing loss enables early recognition of comorbidities such as visual loss in Usher syndrome, potential thyroid issues in Pendred syndrome, and kidney disease in Alport syndrome^6,7^. At least, knowing the etiology can help better inform parents of the possible options and prognosis.

Like this, there have been many reports of the etiologic spectrum using genetic tests or imaging tests, respectively. Non-syndromic and syndromic genetic causes are known to account for about 45% of bilateral SNHL in pediatric patients^8^. Congenital CMV infection (cCMV) reportedly accounts for about 17% of total bilateral SNHL with varying degrees, however this figure could reach upto 33% among those exclusively with an unknown etiology, bringing up the issue of importance of scanning the lesions of central nervous system (CNS) or cochlear enhancement that could suggest cCMV related deafness^8^. DFNB1 due to *GJB2* variants is the most common non-syndromic hearing loss accounting for about 30% of confirmed genetic cases and Pendred, Usher and Waardenburg syndromes are known to be the most common syndromic hearing loss^9^. A genetic study in 459 adult and child CI subjects revealed that top five causative genes were *GJB2* (16%), *TMPRSS3* (10%), *SLC26A4* (8%), *MYO7A* (7%), and *MT-RNR1* (5%)^10^. However, to the best of our knowledge, there is paucity of reports clarifying the etiologic spectrum of pediatric CI candidates manifesting bilateral SP-SNHL by internal auditory canal (IAC)-MRI in combination with state-of-the-art genetic tests including chromosome microarray analysis (CMA) in the single SP-SNHL cohort. Therefore, it was not feasible to compare the relative prevalence of specific imaging abnormalities and molecular genetic abnormalities in the bilateral SP-SNHL group. Further, in previous studies using IAC-MRI, cohorts were mixed with both unilateral and bilateral hearing loss, which precludes these previous analyses from isolating the etiologic diagnostic yield of IAC-MRI to only bilateral pediatric SP-SNHL. Conversely, in most studies addressing the molecular genetic etiologies of pediatric hearing loss patients^11–16^, comprehensive IAC-MRI findings were not provided in tandem, leading to failure in elucidating the full etiologic spectrum in a single cohort.

Recently, our group has identified and published the etiologic spectrum of mild to moderate SNHL in children through state-of-the-art genetic tests^17,18^. While imaging tests contribute relatively little to the elucidation of etiology of mild to moderate SNHL in children, their contribution is much larger in pediatric SP-SNHL. We believed that it was necessary to elucidate the full etiologic spectrum of pediatric SP-SNHL again through combination of genetic tests and imaging tests. Given this, we aim to establish the full etiologic spectrum of pediatric bilateral SP-SNHL by combining both IAC-MRI and state-of-the-art genetic tests, thereby, to the best of our knowledge, providing the highest diagnostic yield of pediatric bilateral SP-SNHL in literature.

## Results

### Overall etiologic spectrum of pediatric SP-SNHL

The etiology of SNHL was convincingly confirmed in 65.5% (78/119) of subjects (Table 1). Following strict criteria for interpretation of pathogenic potential of candidate variants, molecular genetic and radiologic diagnoses were made in 39.4% and 43.7% of subjects, respectively. The genetic tests and IAC-MRI found an overlapping etiology in 17.6% (21/119) of cases. If one of the two tests were performed independently, the etiologic diagnostic rate would be reduced by at least 21.8% without genetic tests, and 26.1% without IAC-MRI.

**Table 1:** Distribution of the pediatric severe-to-profound SNHL subjects, categorized by IAC-MRI findings and Molecular Genetic testing results.

	Abnormal MRI finding (43.7%)	Normal IAC MRI finding (56.3%)	Total
Causative genetic variant confirmed (39.4%)	**n = 21 (17.6%)**	**n = 26 (21.8%)**	N = 47 (39.4%)
Causative genetic variant unconfirmed (60.6%)	**n = 31 (26.1%)**	n = 41 (34.5%)	N = 72 (60.6%)
Total	N = 52 (43.7%)	N = 67 (56.3%)	N = 119

78 subjects (66.5%) (bold) are etiologically diagnosed either by MRI or molecular genetic testing.

### Documented molecular genetic etiology

Molecular genetic testing was omitted in 12 subjects due to identification of non-genetic etiologies either from MRI or additional clinical information (seven subjects with CMV infections, three with cochlear nerve deficiency and one each with diffuse brain atrophy and neonatal intracranial hemorrhage).

Of the 107 subjects who underwent genetic testing, 47 were genetically diagnosed (Tables 1, 2A). The etiology in 26 of these would not be elucidated without genetic testing, as they presented with normal IAC-MRI results (Table 1). The molecular genetic etiologies of the 47 genetically diagnosed subjects were all point variants. The details of the genetic test results are shown in Table 2A and Supplementary Table S1A. *SLC26A4* was the most frequent gene that harbors causative recessive variants in 18 subjects, followed by *GJB2* variants (n = 10) and *OTOF* variants (n = 10). Other causative genes were *TMC1*, *MYO15A*, *NLRP3, ATP1A3*, *KCNQ1, PAX3, POU3F4, PDZD7* and *CHD7* (n = 1 each).

**Table 2:** Molecular genetic etiology of pediatric severe to profound hearing loss in our cohort.

	Most likely causative genetic alteration for hearing loss	No. of subjects
(**A) Genetically confirmed cases by ACMG/AMP guideline (n = 47)**		
Non-syndromic or syndromic single autosomal deafness gene (n = 47)	*SLC26A4*	18
	*GJB2*	10
	*OTOF*	10
	*TMC1*	1
	*MYO15A*	1
	*NLRP3* (CINCA syndrome)	1
	*ATP1A3* (CAPOS syndrome)	1
	*KCNQ1* (Jervell and Lange-Nielsen syndrome)	1
	*PAX3* (Waardenburg syndrome*)	1
	*POU3F4*	1
	*PDZD7*	1
	*CDH7* (CHARGE syndrome**)	1
**(B) Cases diagnosed with candidate variants with high probability, but ACMG/AMP criteria were not satisfied (n = 10)**		
Non-syndromic or syndromic single autosomal deafness gene (n = 7)	*TMC1*	2
	*MYO15A*	1
	*DIAPH1*	1
	*MET*	1
	*MYO6*	1
	*MYO7A*	1
Chromosomal abnormality (n = 3)	Chr. 4p16.3deletion (Wolf–Hirschhorn syndrome)	1
	Chr.18q deletion	1
	Chr. 22q13 deletion	1

Our cohort includes two Waardenburg syndrome subjects*, only one of whom turns out to carry a *PAX3* variant and four CHARGE syndrome subjects**, only one of whom has a *CHD7* variant. The 3q deletion detected from SB318-627 was excluded from the final list due to uncertainty of causality of SNHL.

Additionally, there were 10 subjects who are ‘genetically suspected,” or with candidate variants with high probability of causality (Table 2B, Supplementary Table S1B). With the inclusion of these cases, the diagnostic yield of genetic tests could increase up to 47.9% (57/119). There were also three cases where genetic variants possibly associated with SNHL were identified but the criteria were not satisfied (Supplementary Table S1C).

There were 11 subjects manifesting syndromic deafness, and five of them were identified to carry a causative genetic variant (Table 2A). In the case of CHARGE syndrome (n = 4), only one subject carried a detectable *CDH7* variant; likewise, in the case of Waardenburg syndrome (n = 2), only one subject carried a detectable *PAX3* variant. A causative genetic abnormality was detected from the three other syndromic subjects (CINCA syndrome, CAPOS syndrome and Jervell and Lange-Nielsen syndrome, n = 1 each). In the ‘genetically suspected’ cases, Chromosome 4p16.3 deletion was detected in one subject suspected with Wolf-Hirschhorn syndrome (Table 2B).

None of the four chromosomal deletions detected were able to satisfy the ACMG/AMP criteria. Three subjects with chromosomal deletions (Chr. 4p16.3 deletion, Chr. 18q deletion, and Chr. 22q13.3 deletion) were thus classified as cases with ‘genetically suspected’ variants (Table 2B). However, the association between 3q deletion (SB318-627) and SNHL was doubtful.

### Radiologically documented etiology based on IAC-MRI findings

IAC-MRI revealed radiologic abnormalities that were either direct or indirectly related to SP-SNHL in 52 (43.7%) out of 119 subjects (Tables 1, 3). Among these, pathologies in the inner ear (cochlea/vestibular aqueduct) were the most common, followed by auditory nerve and central nervous system (CNS) lesions (42.3% (22/52) vs 38.5% (20/52) vs 19.2% (10/52), providing a roughly 1:1:0.5 ratio and emphasizing the importance of taking a careful look at the auditory nerve and CNS rather than limiting ourselves to the inner ear.

**Table 3:** Detailed IAC-MRI abnormal findings in our pediatric SP-SNHL cohort.

IAC MRI abnormality	Number (n = 22)
**Inner ear**	
Enlarged vestibular aqueduct With/without incomplete partition type 2 (IP-2)	17
Enlarged vestibular aqueduct With white matter signal intensity abnormality	1
Bilateral cochlear incomplete partition type I (IP-1) With cochlear nerve deficiency	1
Unilateral cochlear incomplete partition type I (IP-1) With contralateral cochlear nerve deficiency With contralateral cochlear aplasia	1
Cochlear incomplete partition type III (IP-3)	1
Active labyrinthitis as documented by enhancement in FLAIR contrast images	1

	(n = 20)
**Cochlear nerve**	
Cochlear nerve deficiency only	13
Cochlear nerve deficiency (CHARGE syndrome) With lateral canal dysplasia and anomalous facial nerve course	4
Cochlear nerve deficiency With cochlear a-/hypo-plasia With thinning of corpus callosum	2
Cochlear nerve deficiency With mild periventricular leukomalacia (Waardenburg IV)	1

	(n = 10)
**Central nervous system lesion (associated with CMV deafness)**	
Periventricular leukomalacia With/without white matter abnormality including polymicrogyria, pachygyria	6
Periventricular leukomalacia With hydrocephalus or enlarged ventricle	2
White matter signal intensity abnormality and polymicrogyria	1
White matter signal intensity abnormality only	1
Total	52

**Figure 1:**
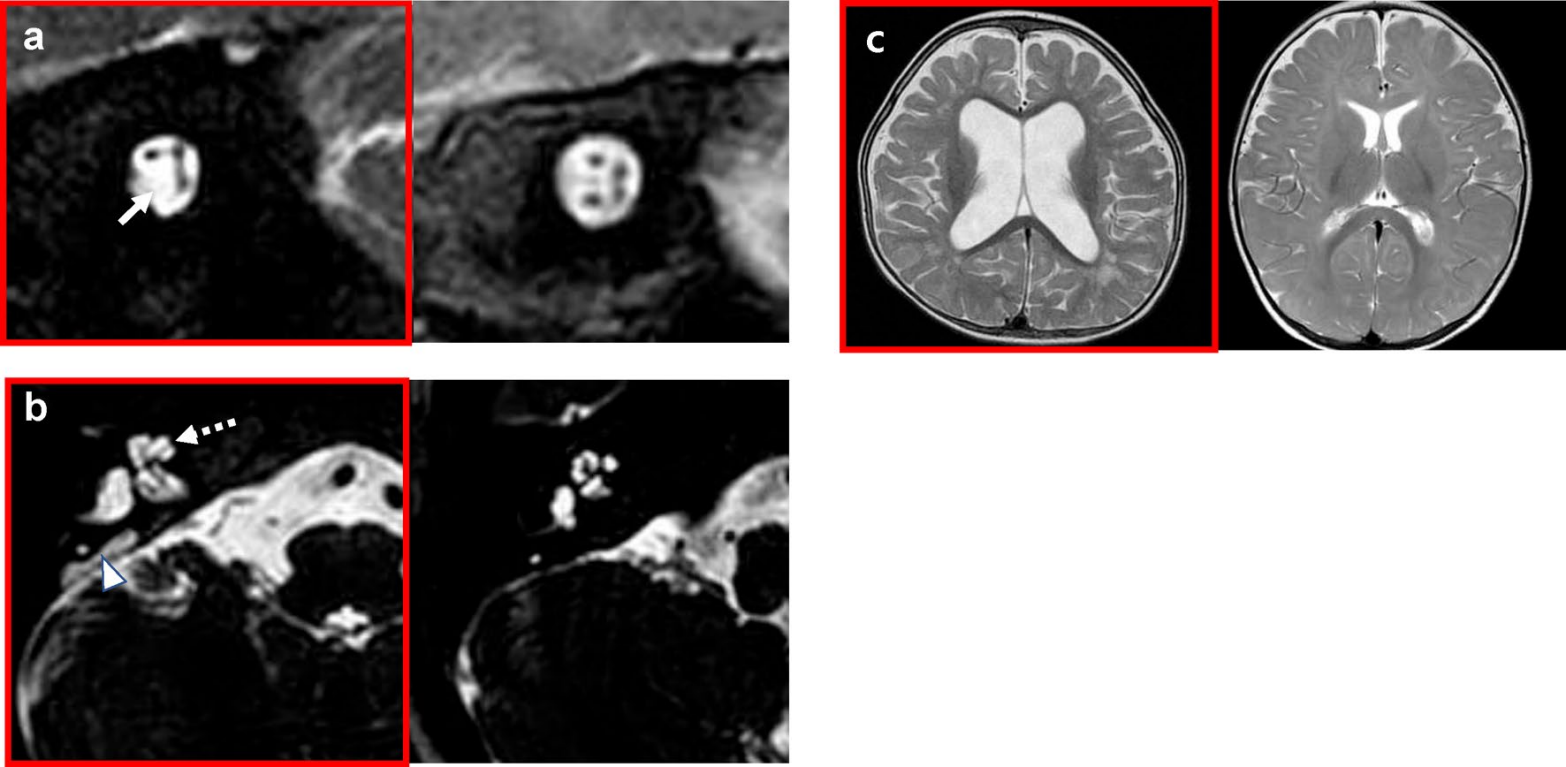
Three major abnormalities related to pediatric SP-SNHL as seen in IAC-MRI. Images on the left side show abnormal findings, while normal findings are on the right. (**a**) Cochlear nerve deficiency: Cochlear nerve is not observed in the internal auditory canal (arrow). (**b**) Enlarged vestibular aqueduct with incomplete partition type 2: Vestibular aqueduct is enlarged and conical in shape (arrowhead), and the interscalar septum is not present (dotted arrow). (**c**) Periventricular leukomalacia: Ventriculomegaly with irregular margins of the bodies of the lateral ventricles and loss of periventricular white matter are observed.

In our pediatric cohort, cochlear nerve deficiency (CND) was the most prevalent radiologic abnormality (n = 20), accounting for 16.8% of our entire cohort, followed by enlarged vestibular aqueduct (EVA) (n = 18 (15.1%)) and periventricular leukomalacia (PVLM) (n = 8 (6.7%)) (Table 3, Fig. 1). CND was detected not only in an idiopathic form, but also in various syndromes, such as CHARGE syndrome (n = 4) and Waardenburg type IV syndrome (n = 1).

In our cohort with 119 subjects, the significant CNS lesions were observed from 19 subjects (16.0%). Notably, CNS lesions found on IAC-MRI were considered related to pediatric SP-SNHL in 10 subjects with documented or strongly suspected congenital CMV infection based on audiological phenotypes, CMV viral culture and CMV-PCR testing, making CNS lesion an important radiological finding in pediatric SP-SNHL (Table 3).

The other nine subjects with CNS lesions consist of four cases with PVLM without any evidence suggesting congenital CMV infection and one each of ‘diffuse brain atrophy’, ‘cystic cerebromalacia’, ‘T2 high signal lesions in both cerebral subcortex’, ‘asymmetric size of lateral ventricle with tinning of corpus callosum’ and ‘T2 high signal lesions in both globus pallidus’ (Supplementary Table S2). In our present study, these MRI abnormalities did not qualify as the etiologic diagnosis of SP-SNHL.

### Interpretation of SP-SNHL without genetic variants or abnormal MRI findings (n = 41)

We suspected that at least five of the 41 subjects might have congenital CMV infection because of either positive CMV culture and CMV-PCR results taken just after 3 weeks (n = 4), or the progressive and asymmetric character of SNHL (n = 1). However, we were not able to confirm the etiology of hearing loss of these subjects as congenital CMV infection.

## Discussion

Herein, we elucidated the etiologic spectrum of 119 prospectively recruited pediatric SP-SNHL subjects and were able to establish a diagnostic rate of 65.5% (78/119) using a protocol that combined IAC-MRI and genetic tests. To the best of our knowledge, this etiologic diagnostic rate in pediatric SP-SNHL subjects is the highest among studies of this type.

Notably, CND turned out to be the most frequent IAC-MRI abnormality (16.8%), even outnumbering EVA. A retrospective study of 207 child patients with bilateral SNHL revealed there were 10 cases (4.8%) of CND and 20 cases (9.7%) of EVA in the cohort^19^. It could be due to the cohort criteria, difference of region and image diagnosis criteria. Recently, a couple of candidate genes that might be associated with CND or other cochlear anomalies have been proposed through a trio study^20^; however, none of these candidate genes were causally linked to pediatric subjects in our cohort. Therefore, taking IAC-MRI from pediatric SP-SNHL patients in a timely manner is of paramount importance.

Detection of CNS lesions such as PVML in IAC-MRI from ten subjects (8.4% of the entire cohort) also supported the etiologic diagnosis among cases with suspected or already diagnosed congenital CMV infection. This figure was not significantly different from 6.6% of severe to profound SNHL previously reported by Lin et al.^21^.

The diagnostic yield of the temporal bone CT had been previously reported to be approximately 18 to 20 percent^21–23^, while very recent studies suggested 30 to 37 percent^24,25^. A similar range was noted for IAC-MRI, ranging from 24 to 42.7%^21,24–27^. However, most of these studies included both bilateral and unilateral SNHL cases in their analysis. The present study is unique in that we included purely bilateral SP-SNHL cases and we were able to achieve a diagnostic yield of 43.7% for IAC-MRI.

Up to 60% of congenital hearing loss is thought to be due to genetic etiologies^28,29^. With the improvement of genetic diagnosis technology and the accumulation of genetic information for hearing loss, the molecular genetic diagnostic rate has been significantly increasing^30,31^. In accordance with this, a total of 12 kinds of monogenic autosomal genes accounted for SP-SNHL in our pediatric cohort. Without extensive use of exome sequencing, *ATP1A3*^32^, *NLRP3*^12,13^, and *PDZD7*^16^ could not have been screened, leading to a decrease in diagnostic rate by 2.5%. In the literature, the diagnostic yield of SNHL with genetic consideration has been reported to be between 19 and 39%^21,23,24,27,33^. The most common causative gene was *GJB2*, accounting for approximately 6–10%—but as high as 20–25% in a certain population—of congenital hearing loss^22,24,27^. Recently, our group reported that 54.8% of pediatric SP-SNHL were genetically diagnosed through rigorous genetic tests^34^. In our present study 47 of 119 (39.5%) subjects were found to have a genetic etiology. This lower diagnostic rate could be attributed to the significantly stricter criteria for inclusion to the ‘genetically diagnosed’ group in our present study. In this study, only the autosomal dominant with ‘pathogenic’ or ‘likely pathogenic’ and the autosomal recessive cases with both ‘likely pathogenic’ variants based on ACMG/AMP guideline^35,36^ were eligible as ‘genetically diagnosed’ cases. If we included the seven cases suspected to have potentially causative genetic variants for calculation of the diagnostic rate, then the proportion of pediatric SP-SNHL subjects with the molecular genetic diagnosis and the etiologic diagnosis could even reach up to 45.3% (54/119) and 71.4% (85/119), respectively. Actually, before the ACMG/AMP guideline was issued, these seven cases would have been considered ‘genetically diagnosed’.

Additionally, it is notable that our study included FISH into the molecular genetic diagnostic battery, thus identifying chromosomal abnormalities that were suspected to have a causal relationship with SP-SNHL in 2.5% (3/119) of the cohort. Although the causality between these three large genomic deletions and SP-SNHL deafness is not fully established due to nonfulfillment of the ACMG/AMP guideline criteria, we believe that these abnormalities are associated with SNHL for the following reasons: Chr.18q deletion was reported to be associated with SNHL^37,38^ and congenital hypothyroidism itself frequently accompanying 18q deletion could also serve as a risk factor for SNHL^39^. PVLM is also a common sign of 18q deletion^40^ and the developmental insult itself causing PVLM could cause hearing loss^41^, also providing causality between 18q deletion and hearing loss. The hemizygous 4p16.3 deletion has also been proposed to be a molecular genetic cause of SNHL^42^ and Wolf–Hirschhorn syndrome^43^. Lastly, an etiologic diagnosis of hearing loss from SB418-819 with a VUS, hemizygous 22q13.3 deletion, mild CND and thinning of the corpus callosum was challenging. However, some children carrying 22q13.3 deletion showed either auditory neuropathy or central auditory processing disorder^44^, which was partially compatible with the presence of mild CND and thinning of corpus callosum. If these three subjects carrying chromosomal deletions that were likely to be associated with SP-SNHL were also included, total diagnostic rates would increase potentially up to 73.9% (88/119).

**Figure 2:**
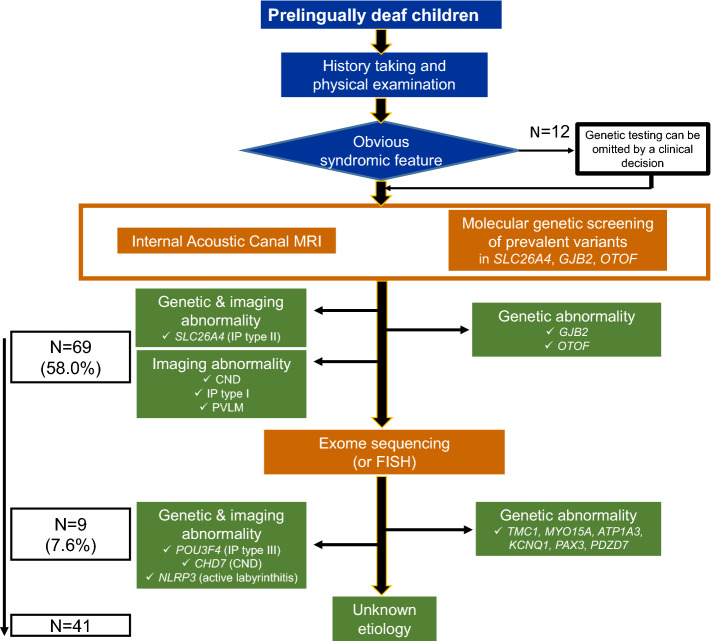
Systematic etiologic diagnostic flowchart used in our study. First line screening using internal auditory canal MRI in tandem with molecular screening of prevalent variants enables etiologic identification in 58.0% of subjects. Subsequent in-depth genetic testing elucidates molecular etiology in 9 more subjects (7.6%), leaving 41 subjects etiologically undiagnosed.

Notably, Lin et al.^21^ pointed out that genetically diagnosed DFNB1 subjects carrying variants in *GJB2* were nearly nonoverlapping compared with children etiologically diagnosed through imaging. This raised the possibility that comprehensive diagnostic battery incorporating both genetic tests and imaging study may significantly improve the etiologic diagnostic rate. However, studies evaluating the combination of genetic tests and imaging study are scarce in literature. Among these studies, the diagnostic yield ranged from 33 to 40%^22,23^. These did not test the genes beyond m.1555A > G, *GJB2* and *SLC26A4,* and also did not report the CNS lesions or CND in detail, leaving room for further investigations. Regardless of the necessity of exome sequencing, the first line screening for prevalent variants of *GJB2*, *OTOF* and *SLC26A4* using previously reported diagnostic kits^45,46^ in tandem with IAC-MRI could possibly lead to better etiologic diagnosis, as high as 58.0% in pediatric SP-SNHL (Fig. 2).

Even with rigorous genetic tests and imaging studies, about 30% of etiology was still not clarified. Undiagnosed cases might be related to perinatal problems such as mostly CMV infection, hypoxemia, hyperbilirubinemia and sepsis, ototoxic medication exposure, autoimmune etiologies and other congenital infections including syphilis, rubella^47–49^. So, in addition to genetic and imaging studies, family and medical history taking, evaluation of comorbidities, neonatal CMV screening and testing for congenital syphilis or rubella should also be considered for evaluation of congenital hearing loss^50^.

Consensus statement of the International Pediatric Otolaryngology Group recommended including genetic tests in etiology study for pediatric bilateral SNHL patients^51^. In the era of precision medicine, this etiologic information could serve as an important basis for decision making in auditory rehabilitation, prediction of prognosis after cochlear implantation, and potentially future gene therapies. Based on what we observed from the present study, the etiologic diagnostic rate would pave the way for future efforts to uncover the hidden causative genes, especially related to CND, and to find diagnostic methods which make it possible to confirm congenital CMV deafness even after the current golden time of diagnosis (within 3 weeks after birth) has passed.

In this study, we have clearly identified the cause of hearing loss in at least 65.5% of pediatric SP-SNHL subjects. Increasing etiologic diagnostic rate of pediatric SP-SNHL is expected to provide the effective hearing rehabilitation and help spread genetic tests in subject with hearing loss. The ratio of abnormal findings on IAC-MRI and genetic tests among our cohort is almost the same, but the overlapping rate is only about 17.6%. So if both tests are not performed, the causative etiology can be missed in more than 20% of patients. Analysis of imaging findings revealed CND as the most common cause among the entire SP-SNHL cohort, and CNS lesions qualifying for diagnostic clues are also observed in 8.4% of subjects. Based on this, it seems necessary to always consider the possibility of CND or CNS lesions when interpreting IAC-MRI findings.

## Methods

### Recruitment of study participants

We prospectively established a cohort exclusively comprised of children under the age of 15 years, who were all treated by a single surgeon (B.Y.C) in the same tertiary referral hospital between May 2013 and September 2020 for SP-SNHL. In total, 119 patients meeting the criteria of bilateral SP-SNHL with 500, 1000 and 2000 Hz averaging hearing thresholds exceeding 70 dB HL were included. Subjects with single sided deafness or asymmetric hearing loss were excluded. This study was carried out in accordance with the Declaration of Helsinki. The study was approved by the Seoul National University Bundang Hospital Institutional Review Board (IRB Number B-2108-705-103). All participants and their legal guardians were given written informed consent before participating in this study.

### Data collection

Demographic data, IAC-MRI findings, and genetic test results were collected and analyzed^52^. The mean age of children at recruitment after rigorous evaluation of hearing thresholds was 27.96 months (± 27.432, 6–178 months). Male-to-female ratio of subjects was 75:44. IAC-MRI was carried out in all 119 cases. Abnormal IAC-MRI findings related to SP-SNHL were divided into those from the inner ear, the cochlear nerve, and the central nervous system (CNS). If the IAC-MRI results were consistent with the syndrome with SP-SNHL as one of the symptoms, it was determined to account for the SP-SNHL.

At least one of the four genetic tests: U-Top screening kit^45,46^, panel sequencing^53,54^, exome sequencing^18,55^ or fluorescent in situ hybridization (FISH), was carried out in 107 subjects. For those without any confirmatory molecular diagnosis by U-Top screening kit, panel sequencing or exome sequencing were performed. Unless panel sequencing clearly revealed the causative variants, exome sequencing was performed. FISH was performed only for syndromic deafness with significant co-morbid abnormality, of which causative variants had not been elucidated after exome sequencing^56^. In all cases, genomic DNA was extracted from the peripheral blood. Obtained reads from panel or exome sequencing were mapped onto the University of California–Santa Cruz (UCSC) hg19 reference genome assembly, using the Lasergene 14 software package (DNASTAR, Madison, WI, USA). Several global minor allele frequency (MAF) databases were consulted for checking minor allele frequency, such as Exome Aggregation Consortium (ExAC), 1000 Genomes Project (1000 Genomes), National Heart, Lung, and Blood Institute (NHLBI), as well as the Grand Opportunity Exome Sequencing Project (GO-ESP) and the Korean Reference Genome Database (KRGDB). Single-nucleotide polymorphisms (SNP) that were incompatible with the autosomal recessive pattern were ruled out. To predict the pathogenicity of each missense variant, we referred to diverse in silico prediction software, such as Sorting Intolerant from Tolerant (SIFT), PolyPhen-2, MutationTaster, Combined Annotation Dependent Depletion (CADD), and Rare Exome Variant Ensemble Learner (REVEL) analyses. Whenever possible, the remaining SNPs were validated in other family members by Sanger sequencing for segregation study.

Description of pathogenic potential of candidate variants were made according to the American College of Medical Genetics and Genomics (ACMG) 2015 guidelines, the newly specified ACMG/Association for Molecular Pathology (AMP) hearing loss rules^36^, and ‘Expert specification of the ACMG/AMP variant interpretation guidelines for genetic hearing loss’^35^. In autosomal recessive (AR) cases, it was notated as ‘genetically diagnosed’ only when both variants were classified as at least ‘likely pathogenic (LP)’. In autosomal dominant (AD) cases, it was indicated as ‘genetically diagnosed’ when there was a variant of ‘LP’ or ‘pathogenic’ classification. Cases with candidate genetic variants of AD inheritance classified as ‘variant of unknown significance (VUS)’ and of AR inheritance with one variant as ‘LP’ or ‘pathogenic’ but the other variant as ‘VUS’ were notated as ‘genetically suspected’. When a large genomic deletion with high probability of relationship with deafness was identified but not 100% certain, it was also notated as ‘genetically suspected’. Notably, genetic tests were omitted in 12 cases where etiological diagnosis had already been established through IAC-MRI.

### Diagnostic yield of each modality, categorization of the subjects

The diagnostic yield of IAC-MRI and genetic tests for our pediatric SP-SNHL cohort was calculated. Subjects with ‘genetically suspected’ candidate variants were not considered ‘genetically diagnosed’.

## Supplementary Information

Supplementary Tables.

## Data Availability

The data that support the findings of this study are available from the corresponding author upon reasonable request. Some data may not be made available because of privacy or ethical restrictions.
