# Supplementary material for: Full etiologic spectrum of pediatric severe to profound hearing loss of consecutive 119 cases
Source: Sci Rep. 2022 Jul 19;12:12335. doi: 10.1038/s41598-022-16421-x (PMC9296524; doi:10.1038/s41598-022-16421-x)
Supplement: Supplementary file 1 — Supplementary Tables. [file 41598_2022_16421_MOESM1_ESM.pdf]

Supplementary Table S1. Details of genotypes from subjects with candidate variants

A) Genetically confirmed cases by ACMG/AMP guideline **(n=47)**

Sample ID	Gene Name<br>NM/ MP	Inheritance	Variant<br>Genomic Position<br>dbSNP ID	Zygosity	Prediction Algorithm			MAF		Classification of pathogenic variants
					CADD PHRED	REVEL SCORE	MaxEntScan	Global MAF	KRGDB	
SB129-222	SLC26A4<br>NM_000441.2<br>NP_000432.1	AR	c.919-2A>G<br>chr7:107323898<br>rs111033313	heterozygous	34	NA	WT:9.25<br>MT:1.29	G=0.0003/37<br>(ExAC)	0.000873	**Pathogenic**<br>PVS1, PM3_VS, PP1_S
			c.2168A>G:p.His723Arg<br>chr7:107350577<br>rs121908362	heterozygous	25.2	0.933	NA	G=0.000124/15<br>(ExAC)	0.005824	**Pathogenic**<br>PM2_P, PM3_VS, PS1, PP3
SH143-298	SLC26A4<br>NM_000441.2<br>NP_000432.1	AR	c.2168A>G:p.His723Arg<br>chr7:107350577<br>rs121908362	heterozygous	25.2	0.933	NA	G=0.000124/15<br>(ExAC)	0.005824	**Pathogenic**<br>PM2_P, PM3_VS, PS1, PP3
			c.1174A>T:p.Asn392Tyr<br>chr7:107330593<br>rs201562855	heterozygous	28.5	0.981	NA	T=0.000008 (1/121330, ExAC)	NA	**Pathogenic**<br>PM2, PM3_VS, PP3,
SB204-398	OTOF<br>NM_001287489.2<br>NP_001274418.1	AR	c.5816G>A:p.Arg1939Gln<br>chr2:26683866<br>rs80356605	heterozygous	25.1	0.86	NA	T=0.00003/1 (ExAC)	T=0.001452/5	**Pathogenic**<br>PS3, PM1, PM2, PM3_VS, PP3, PP4
			c.5566C>T:p.Arg1856Trp<br>chr2:26681086<br>rs368155547	heterozygous	26.4	0.723	NA	A=0.00004/5 (ExAC)	A=0.000871/3	**Pathogenic**<br>PS4, PM2, PM3, PP1, PP3, PP4
			c.4227+5G>C<br>chr2:26690228<br>rs571671530	heterozygous	12.99	NA	WT:8.68<br>MT:5.19	G=0.000058/7<br>(ExAC)	0.000294	**Likely pathogenic**<br>PM2, PM3, PP1, PP3, PP4
SB214-417	SLC26A4<br>NM_000441.2<br>NP_000432.1	AR	c.919-2A>G<br>chr7:107323898<br>rs111033313	homozygote	34	NA	WT:9.25<br>MT:1.29	G=0.0003/37<br>(ExAC)	0.000873	**Pathogenic**<br>PVS1, PM3_VS, PP1_S
SB333-656	NLRP3<br>NM_001243133.1<br>NP_001230062.1	AD	c.1985T>C:p.Met662Thr<br>chr1:247588736<br>rs180177435	heterozygous	17.87	0.494	NA	NA	NA	**Likely pathogenic**<br>PS2, PM2
SB004-011	OTOF<br>NM_001287489.2<br>NP_001274418.1	AR	c.3192C>G:p.Tyr1064*<br>chr2:26697477rs766819324	heterozygous	38	NA	NA	C=0.000008<br>(1/119874, ExAC)	C=0.000292/1	**Pathogenic**<br>PVS1_VS, PM2, PM3_S, PP1_M, PP4
			c.5203del:p.Arg1735Glyfs*28<br>rs727503352	heterozygous	36	NA	NA	delG=0.000008<br>(1/121144, ExAC)	NA	**Pathogenic**<br>PVS1_VS, PM2, PM3_S, PP1_M, PP4
SH349-770	GJB2<br>NM_004004.5<br>NP_003995.2	AR	c.235delC:p.Leu79Cysfs*3<br>chr13:20763488<br>rs80338943	homozygous	32	NA	NA	delG=0.000363<br>(44/121376, ExAC)	NA	**Pathogenic**<br>PVS1, PS3_M, PM1, PM3_VS
SB239-463	OTOF<br>NM_001287489.2<br>NP_001274418.1	AR	c.3032T>C :p.Leu1011Pro<br>Chr2:26698321<br>rs80356596	heterozygous	29.5	0.902	NA	NA	NA	**Pathogenic**<br>PM2, PM3_VS, PP1, PP3
			c.2521G>A:p.Glu841Lys<br>chr2:26700042<br>rs772729658	heterozygous	24	0.745	NA	T=0.000029<br>(3/102728, ExAC)	NA	**Pathogenic**<br>PS4, PM2, PM3, PP1, PP3, PP4
SB239-464	OTOF<br>NM_001287489.2<br>NP_001274418.1	AR	c.5816G>A:p.Arg1939Gln<br>chr2:26683866<br>rs80356605	heterozygous	25.1	0.760	NA	T=0.00013<br>T=0.00003/1 (ExAC)	T=0.001452/5	**Pathogenic**<br>PS4, PM2, PM3, PP1, PP3, PP4

			c.2521G>A:p.Glu841Lys<br>chr2:26700042<br>rs772729658	heterozygous	24	0.745	NA	T=0.000029<br>(3/102728, ExAC)	NA	**Pathogenic**<br>PS4, PM2, PM3, PP1, PP3, PP4
SB281-553	TMC1<br>NM_138691.2<br>NP_619636	AR	c.100C>T:p.Arg34*<br>chr9:75309494<br>rs121908073	heterozygous	34	NA	NA	T=0.00005/6<br>(ExAC)	NA	**Pathogenic**<br>PVS1, PM2, PP1_S
			c.1250G>A:p.Gly417Glu<br>chr9:75406827<br>dbSNP ID: NA	heterozygous	26.3	0.557	NA	NA	NA	**Likely pathogenic**<br>PM2, PM3_PM5
SH234-547	OTOF<br>NM_001287489.2<br>NP_001274418.1	AR	c.5816G>A:p.Arg1939Gln<br>chr2:26683866<br>rs80356605	heterozygous	25.1	0.86	NA	T=0.00003/1 (ExAC)	T=0.001452/5	**Pathogenic**<br>PS3, PM1, PM2, PM3_VS, PP3, PP4
			c.5566C>T:p.Arg1856Trp<br>chr2:26681086<br>rs368155547	heterozygous	26.4	0.723	NA	A=0.00004/5 (ExAC)	A=0.000871/3	**Pathogenic**<br>PS4, PM2, PM3 PP1, PP3, PP4
SH222-518	ATP1A3<br>NM_001256214<br>NP_001243143	AD	c.2491G>A:p.Glu831Lys<br>chr19:42474427<br>rs587777771	heterozygote	25	0.967	NA	NA	NA	**Pathogenic**<br>PS2_VS, PM1, PM2, PP1_S, PP3
SB1-1	SLC26A4<br>NM_000441.2<br>NP_000432.1	AR	c.2168A>G:p.His723Arg<br>chr7:107350577<br>rs121908362	homozygous	25.2	0.933	NA	G=0.000124/15<br>(ExAC)	0.005824	**Pathogenic**<br>PM2_P, PM3_VS, PS1, PP3
SB357-698	SLC26A4<br>NM_000441.2<br>NP_000432.1	AR	c.2027T>A:p.Leu676Gln<br>chr7:107342495<br>rs111033318	heterozygous	26.3	0.733	NA	A=0.000024<br>(3/125568, TOPMED)	0.000874	**Pathogenic**<br>PM2, PM3_VS, PP3
			c.2168A>G:p.His723Arg<br>chr7:107350577<br>rs121908362	heterozygous	25.2	0.933	NA	G=0.000124/15<br>(ExAC)	0.005824	**Pathogenic**<br>PM2_P, PM3_VS, PS1, PP3
SB367-718	GJB2<br>NM_004004.5<br>NP_003995.2	AR	c.235delC:p.Leu79Cysfs*3<br>chr13:20763488<br>rs80338943	heterozygous	32	NA	NA	GG=0.000363/44<br>(ExAC)	NA	**Pathogenic**<br>PVS1, PS3_M, PM1, PM3_VS
			c.427C>T:p.Arg143Trp<br>chr13:20763292<br>rs80338948	heterozygous	29.3	0.918	NA	A=0.000165<br>(20/121050, ExAC)	0.000582	**Pathogenic**<br>PM2_P, PM3_VS, PM5, PP3
SB359-702	GJB2<br>NM_004004.5<br>NP_003995.2	AR	c.109G>A:p.V37I<br>chr13:20763612<br>rs72474224	heterozygous	21.7	0.656	NA	T=0.006587/799 (ExAC)	0.006407	**Pathogenic**<br>PS4, PM1, PM3, PM5, PP1_S
			427C>T:p.Arg143Trp<br>chr13:20763292<br>rs80338948	heterozygous	29.3	0.918	NA	A=0.000165<br>(20/121050, ExAC)	0.000582	**Pathogenic**<br>PM2_P, PM3_VS, PM5, PP3
SB394-761	PDZD7<br>NM_001195263.1<br>NP_001182192.1	AR	c.1669delC:p.Arg557fs*13<br>chr10:102775472<br>dbSNP: NA	heterozygous	NA	NA	NA	NA	NA	**Likely Pathogenic**<br>PVS1, PM2
			c.490C>T:p.Arg164Trp<br>chr10:102783245<br>rs200664140	heterozygous	24.3	0.377	NA	A=0.00005/6<br>(ExAC)	0.003205	**Likely Pathogenic**<br>PM1, PM2, PM3_S, PP1
SB359-702	GJB2<br>NM_004004.5<br>NP_003995.2	AR	c.109G>A:p.V37I<br>chr13:20763612<br>rs72474224	heterozygous	21.7	0.656	NA	T=0.006587/799 (ExAC)	0.006407	**Pathogenic**<br>PS4, PM1, PM3, PM5, PP1_S
			427C>T:p.Arg143Trp<br>chr13:20763292<br>rs80338948	heterozygous	29.3	0.918	NA	A=0.000165<br>(20/121050, ExAC)	0.000582	**Pathogenic**<br>PM2_P, PM3_VS, PM5, PP3
SB430-834	chrXp5 Deletion (POU3F4)									
SB337-668	SLC26A4<br>NM_000441.2<br>NP_000432.1	AR	c.1229C>T:p.Thr410Met<br>chr7:107330648<br>rs111033220	heterozygous	25.2	0.907	NA	T=0.000190 (23/121222, ExAC)	NA	**Pathogenic**<br>PM2, PM3_VS, PP3

			c.2027T>A:p.Leu676Gln<br>chr7:107342495<br>rs111033318	heterozygous	26.3	0.733	NA	A=0.000024 (3/125568,<br>TOPMED)	0.000874	Pathogenic<br>PM2, PM3_VS, PP3
SB455-883	OTOF<br>NM_001287489.2<br>NP_001274418.1	AR	c.2521G>A:p.Glu841Lys<br>chr2:26700042<br>rs772729658	heterozygous	24	0.745	NA	T=0.000029<br>(3/102728, ExAC)	NA	Pathogenic<br>PS4, PM2, PM3, PP1,<br>PP3, PP4
			c.4227+5G>C<br>chr2:26690228<br>rs571671530	heterozygous	12.99	NA	WT:8.68<br>MT:5.19	G=0.000058/7<br>(ExAC)	0.000342	Likely pathogenic<br>PM2, PM3 PP1, PP3, PP4
			c.5566C>T:p.Arg1856Trp<br>chr2:26681086<br>rs368155547	heterozygous	26.4	0.723	NA	A=0.00004/5 (ExAC)	A=0.000871/3	Pathogenic<br>PS4, PM2, PM3 PP1,<br>PP3, PP4
No sample ID	SLC26A4<br>NM_000441.2<br>NP_000432.1	AR	c.919-2A>G<br>chr7:107323898<br>rs111033313	heterozygous	34	NA	WT:9.25<br>MT:1.29	G=0.0003/37<br>(ExAC)	0.000873	Pathogenic<br>PVS1, PM3_VS, PP1_S
			c.2168A>G:p.His723Arg<br>chr7:107350577<br>rs121908362	heterozygous	25.2	0.933	NA	G=0.000124/15<br>(ExAC)	0.005824	Pathogenic<br>PM1, PM2_P, PM3_VS,<br>PS1, PP3
SB371-722	SLC26A4<br>NM_000441.2<br>NP_000432.1	AR	c.2168A>G:p.His723Arg<br>chr7:107350577<br>rs121908362	heterozygous	25.2	0.933	NA	G=0.000124/15<br>(ExAC)	0.005824	Pathogenic<br>PM1, PM2_P, PM3_VS,<br>PS1, PP3
			c.919-2A>G<br>chr7:107323898<br>rs111033313	heterozygous	34	NA	WT:9.25<br>MT:1.29	G=0.0003/37<br>(ExAC)	0.000873	Pathogenic<br>PVS1, PM3_VS, PP1_S
No sample ID	GJB2<br>NM_004004.5<br>NP_003995.2	AR	427C>T:p.Arg143Trp<br>chr13:20763292<br>rs80338948	heterozygous	29.3	0.918	NA	A=0.000165<br>(20/121050, ExAC)	0.000582	Pathogenic<br>PM2_P, PM3_VS, PM5,<br>PP3
			c.235delC:p.Leu79Cysfs*3<br>chr13:20763488<br>rs80338943	heterozygous	32	NA	NA	GG=0.000363/44<br>(ExAC)	NA	Pathogenic<br>PVS1, PS3_M, PM1,<br>PM3_VS
SB464-901	GJB2<br>NM_004004.5<br>NP_003995.2	AR	c.235delC:p.Leu79Cysfs*3<br>chr13:20763488<br>rs80338943	heterozygous	32	NA	NA	GG=0.000363/44<br>(ExAC)	NA	Pathogenic<br>PVS1, PS3_M, PM1,<br>PM3_VS
			c.299_300delAT<br>:p.His100Argfs*14<br>chr13:20763421_20763422del<br>rs111033204	heterozygous	29	NA	NA	delAT=0.000041<br>(5/121252, ExAC)	NA	Pathogenic<br>PVS1, PM2, PM3_S
SH230-538	OTOF<br>NM_001287489.2<br>NP_001274418.1	AR	c.4227+5G>C<br>chr2:26690228<br>rs571671530	heterozygous	12.99	NA	WT:8.68<br>MT:5.19	G=0.000058 (7/121370, ExAC)	0.000294	Likely pathogenic<br>PM2, PM3 PP1, PP3, PP4
			c.2521G>A:p.Glu841Lys<br>chr2:26700042<br>rs772729658	heterozygous	24	0.745	NA	T=0.000029<br>(3/102728, ExAC)	NA	Pathogenic<br>PS4, PM2, PM3, PP1,<br>PP3, PP4
SB493-945	SLC26A4<br>NM_000441.2<br>NP_000432.1	AR	c.919-2A>G<br>chr7:107323898<br>rs111033313	heterozygous	34	NA	WT:9.25<br>MT:1.29	G=0.0003/37<br>(ExAC)	0.000873	Pathogenic<br>PVS1, PM3_VS, PP1_S
			c.2168A>G:p.His723Arg<br>chr7:107350577<br>rs121908362	heterozygous	25.2	0.933	NA	G=0.000124/15<br>(ExAC)	0.005824	Pathogenic<br>PM2_P, PM3_VS, PS1,<br>PP3
SB470-913	KCNQ1<br>NM_000218.2<br>NP_000209.2	AR	c.94A>T:p.Lys32*<br>chr11:2466422<br>rs1554958043	heterozygous	36	NA	NA	T=0.0006<br>(1/1756, Korea1K)	NA	Pathogenic<br>PVS1, PM2_P
			c.1032_1117dup<br>:p.Ser373Trpfs*10<br>chr11:2604775_2604860<br>dbSNP ID: NA	heterozygous	NA	NA	NA	NA	NA	Pathogenic<br>PVS1, PM2, PM3
SB487-939	PAX3<br>NM_181459.3<br>NP_852123.1	AD	c.879dup:p.Phe294Valfs*116<br>chr2:222221306<br>rs1553572967	heterozygous	NA	NA	NA	NA	NA	Likely Pathogenic<br>PVS1, PM2,

**No sample ID**	**SLC26A4 NM_000441.2 NP_000432.1**	**AR**	**c.919-2A>G chr7:107323898 rs111033313**	**homozygous**	**34**	**NA**	**WT:9.25 MT:1.29**	**G=0.0003/37 (ExAC)**	**0.000873**	**Pathogenic** PVS1, PM3_VS, PP1_S
**No sample ID**	**SLC26A4 NM_000441.2 NP_000432.1**	**AR**	**c.2168A>G:p.His723Arg chr7:107350577 rs121908362**	**homozygous**	**25.2**	**0.933**	**NA**	**G=0.000124/15 (ExAC)**	**0.005824**	**Pathogenic** PM2_P, PM3_VS, PS1, PP3
**SB574-1061**	**CDH7 NM_017780.4 NP_060250.2**	**AD**	**c:7879C>T:p.Arg2627* chr8:61774803 rs1064793346**	**heterozygous**	**42**	**NA**	**NA**	**NA**	**NA**	**Pathogenic** PVS1, PM2, PS4_M, PP1
**SB524-992**	**SLC26A4 NM_000441.2 NP_000432.1**	**AR**	**c.1229C>T:p.Thr410Met chr7:107330648 rs111033220**	**homozygous**	**25.2**	**0.907**	**NA**	**T=0.000190 (23/121222, ExAC)**	**NA**	**Pathogenic** PM2, PM3_VS, PP3
**No sample ID**	**MYO15A NM_016239.3 NP_057323.3**	**AR**	**c.8084_8085delAA:p.Lys2695fs chr17:18057206_18057207del rs781143752**	**heterozygous**	**NA**	**NA**	**NA**	**delAA=0.000008 (1/119334, ExAc)**	**NA**	**Likely Pathogenic** PVS1, PM2
			**c.10250_10252delCCT :p.Ser3417del chr17:18075504_18075506 rs760069953**	**heterozygous**	**19.61**	**NA**	**NA**	**delCCT=0.000033 (4/120678, ExAc)**	**NA**	**Likely pathogenic** PM2, PM3, PM4
**No sample ID**	**SLC26A4 NM_000441.2 NP_000432.1**	**AR**	**c.2168A>G:p.His723Arg chr7:107350577 rs121908362**	**heterozygous**	**25.2**	**0.933**	**NA**	**G=0.000124/15 (ExAC)**	**0.005824**	**Pathogenic** PM2_P, PM3_VS, PS1, PP3
			**c.2162C>T;p.Thr721Met chr7:107350571 rs121908363**	**heterozygous**	**25.5**	**0.864**	**NA**	**T=0.000058 (1/121120, ExAC)**	**NA**	**Pathogenic** PM2, PM3_VS, PP3
**SB469-910**	**SLC26A4 NM_000441.2 NP_000432.1**	**AR**	**c.2168A>G:p.His723Arg chr7:107350577 rs121908362**	**homozygous**	**25.2**	**0.933**	**NA**	**G=0.000124/15 (ExAC)**	**0.005824**	**Pathogenic** PM1, PM2_P, PM3_VS, PS1, PP3
**SB469-910**	**SLC26A4 NM_000441.2 NP_000432.1**	**AR**	**c.2168A>G:p.His723Arg chr7:107350577 rs121908362**	**homozygous**	**25.2**	**0.933**	**NA**	**G=0.000124/15 (ExAC)**	**0.005824**	**Pathogenic** PM2_P, PM3_VS, PS1, PP3
**SB543-1015**	**OTOF NM_001287489.2 NP_001274418.1**	**AR**	**c.5816G>A:p.Arg1939Gln chr2:26683866 rs80356605**	**homozygous**	**25.1**	**0.86**	**NA**	**T=0.00003/1 (ExAC)**	**T=0.001452/5**	**Pathogenic** PS3, PM1, PM2, PM3_VS, PP3, PP4
**S537-1009**	**OTOF NM_001287489.2 NP_001274418.1**	**AR**	**c.5203del:p.Arg1735Glyfs*28 chr2:26685039 rs727503352**	**heterozygous**	**NA**	**NA**	**NA**	**delG=0.000008 (1/121144, ExAC)**	**NA**	**Pathogenic** PVS1, PM2, PM3_S, PP4
			**c.2866+5G>A chr2:26698991 rs1572426855**	**heterozygous**	**26.2**	**NA**	**WT:8.28 MT:-1.27**	**NA**	**NA**	**Likely pathogenic** PM2, PM3, PP3, PP4
**No sample ID**	**GJB2 NM_004004.5 NP_003995.2**	**AR**	**c.299_300delAT :p.His100Argfs*14 chr13:20763421_20763422del rs111033204**	**heterozygous**	**29**	**NA**	**NA**	**delAT=0.000041 (5/121252, ExAC)**	**NA**	**Pathogenic** PVS1, PM2, PM3_S
			**427C>T:p.Arg143Trp chr13:20763292 rs80338948**	**heterozygous**	**29.3**	**0.918**	**NA**	**A=0.000165 (20/121050, ExAC)**	**0.000582**	**Pathogenic** PM2_P, PM3_VS, PM5, PP3
**SH346-765**	**OTOF NM_001287489.2 NP_001274418.1**	**AR**	**c.3192C>G: p.Tyr1064* chr2:26697477 rs766819324**	**heterozygous**	**37**	**NA**	**NA**	**C=0.000008 (1/119874, ExAC)**	**C=0.00029/1**	**Pathogenic** PVS1, PM2, PP1, PM3_S PP4
			**c.5203del:p.Arg1735Glyfs*28 chr2:26685039 rs727503352**	**heterozygous**	**NA**	**NA**	**NA**	**delG=0.000008 (1/121144, ExAC)**	**NA**	**Pathogenic** PVS1, PM2, PM3_S, PP4
**SB568-1046**	**SLC26A4 NM_000441.2 NP_000432.1**	**AR**	**c.2168A>G:p.His723Arg chr7:107350577 rs121908362**	**homozygous**	**25.2**	**0.933**	**NA**	**G=0.000124/15 (ExAC)**	**0.005824**	**Pathogenic** PM2_P, PM3_VS, PS1, PP3
**SB599-1097**	**SLC26A4 NM_000441.2 NP_000432.1**	**AR**	**c.2168A>G:p.His723Arg chr7:107350577 rs121908362**	**homozygous**	**25.2**	**0.933**	**NA**	**G=0.000124/15 (ExAC)**	**0.005824**	**Pathogenic** PM2_P, PM3_VS, PS1, PP3

No sample ID	SLC26A4<br>NM_000441.2<br>NP_000432.1	AR	c.2027T>A:p.Leu676Gln<br>chr7:107342495<br>rs111033318	heterozygous	26.3	0.733	NA	A=0.000024 (3/125568, TOPMED)	0.000874	Pathogenic<br>PM2, PM3_VS, PP3
			c.2168A>G:p.His723Arg<br>chr7:107350577<br>rs121908362	heterozygous	25.2	0.933	NA	G=0.000124/15<br>(ExAC)	0.005824	Pathogenic<br>PM2_P, PM3_VS, PS1, PP3
SH130-270	GJB2<br>NM_004004.5<br>NP_003995.2	AR	c.257C>G:p.Thr86Arg<br>chr13:20763464<br>rs1291519904	heterozygous	26.9	0.969	NA	C=0.0005<br>(1/1832, Korea1K)	NA	Pathogenic<br>PM3_VS, PP1, PP3,
			427C>T:p.Arg143Trp<br>chr13:20763292<br>rs80338948	heterozygous	29.3	0.918	NA	A=0.000165<br>(20/121050, ExAC)	0.000582	Pathogenic<br>PM2_P, PM3_VS, PM5, PP3
SB251-495	GJB2<br>NM_004004.5<br>NP_003995.2	AR	c.257C>G:p.Thr86Arg<br>chr13:20763464<br>rs1291519904	heterozygous	26.9	0.969	NA	C=0.0005<br>(1/1832, Korea1K)	NA	Pathogenic<br>PM3_VS, PP1, PP3,
			c.508_511dupAACG<br>:p.Ala171Glufs*40<br>chr13:20763210<br>rs773528125	heterozygous	23.8	NA	NA	dupCGTT=0.000017<br>(2/121034, ExAC)	NA	Pathogenic
SB492-944	GJB2<br>NM_004004.5<br>NP_003995.2	AR	c.257C>G:p.Thr86Arg<br>chr13:20763464<br>rs1291519904	heterozygous	26.9	0.969	NA	C=0.0005<br>(1/1832, Korea1K)	NA	Pathogenic<br>PM3_VS, PP1, PP3,
			c.176_191delGCTGCAAGAACG<br>TGTG: p.Gly59Alafs*18<br>chr13:20763531_20763546del<br>rs750188782	heterozygous	NA	NA	NA	delACACGTTCTTGCAGCC<br>=0.000016 (2/121366, ExAC)	NA	Pathogenic<br>PVS1, PM2,

B) Cases where the probability of hearing loss due to the candidate variants is very high, but the criteria do not satisfy the ACMG criteria **(n=10).**

Sample ID	Gene Name<br>NM/ MP	Inheritance	Variant<br>Genomic Position<br>dbSNP ID	Zygosity	Prediction algorithm			MAF		Classification<br>of pathogenic<br>variants
					CADD<br>PHRED	REVEL<br>SCORE	MaxEntScan	Global MAF	KRGDB	
SB156-272	MYO15A<br>NM_016239.3<br>NP_057323.3	AR	c.3871C>T:p.Leu1291Phe<br>chr17:18030109<br>rs1391331735	heterozygous	24.4	0.74	NA	T=0.000008 (1/125568, TOPMED)	0.000342	VUS<br>PM2, PM3, PP3
			c.5835T>G:p.Tyr1945*<br>chr17:18046079<br>dbSNP ID: NA	heterozygous	38	NA	NA	NA	NA	Likely Pathogenic<br>PVS1, PM2
SB206-400	DIAPH1<br>NM_005219.5<br>NP_005210.3	AD	c.793G>T:p.Ala265Ser<br>chr5:140960342<br>dbSNP ID: NA	heterozygous	25.8	0.786	NA	NA	NA	VUS<br>PM2, PP3, PS3_P
SB276-544	TMC1<br>NM_138691.2<br>NP_619636	AR	c.100C>T:p.Arg34*<br>chr9:75309494<br>rs121908073	heterozygous	34	NA	NA	T=0.00005/6<br>(ExAC)	NA	Pathogenic<br>PVS1, PM2, PM3, PP1_S
			c.1133G>A:p.Gly378Glu<br>chr9:75404142<br>dbSNP ID: NA	heterozygous	23.8	0.69	NA	NA	NA	VUS<br>PM2, PM3
SB429-833	TMC1<br>NM_138691.2<br>NP_619636.2	AR	c.545G>T:p.Gly182Val<br>chr9:75366775<br>rs199560971	heterozygous	27.9	0.885	NA	A=0.000016<br>(2/121388, ExAC)	NA	Likely Pathogenic<br>PM2, PM3_S, PM5, PP3
			c.884C>T:p.Ala295Val<br>chr9:75387471<br>rs760785189	heterozygous	23.1	0.047	NA	NA	NA	VUS<br>PM2, PM3

SB290-582	MYO6<br>NM_004999.3<br>NP_004990	AD	c.667G>A:p.Gly223Arg<br>Chr6:76550946<br>dbSNP ID: NA	heterozygote	29.8	0.923	NA	NA	NA	VUS<br>PM2, PP3
SB540-1012	MET<br>NM_001127500.1<br>NP_001120972.1	AR	c.218T>A:p.Leu73*<br>chr7:116339356<br>dbSNP ID: NA	heterozygous	35	NA	NA	NA	NA	Likely Pathogenic<br>PVS1, PM2
			c.2569C>G:p.Pro857Ala<br>chr7:116403254<br>dbSNP ID: NA	heterozygous	24.9	0.717	NA	NA	NA	VUS<br>PM2, PM3, PP3
SB579-1069	MYO7A<br>NM_000260.3<br>NP_000251.3	AR	c.1431T>A:p.Tyr477*<br>chr11:76873253<br>dbSNP ID: NA	heterozygous	26.2	NA	NA	NA	NA	Likely Pathogenic<br>PVS1, PM2
			c.3560G>T:p.Ser1187Ile<br>chr11:76900445<br>rs1555090314	heterozygous	27.4	0.926	NA	NA	NA	VUS<br>PM2, PM3, PP3
SB418-819	MYH14<br>NM_001145809.1<br>NP_001139281.1	AD	c.3295G>T:p.Asp1099Tyr<br>chr19:50775229<br>rs1600994287	heterozygous	34	0.717	NA	T=0.0005<br>(1/1832, Korea1K)	NA	VUS<br>PP3
					chr22q13 deletion					
No sample ID					chr4p16.3 deletion					
No sample ID					chr18q21.3 deletion					

C) Disqualified cases from a 'genetically diagnosed' group **(n=3)**

Sample ID	Gene Name<br>NM/ MP	Inheritance	Variant<br>Genomic Position<br>dbSNP ID	Zygosity	Prediction algorithm			MAF		Classification<br>of pathogenic<br>variants
					CADD<br>PHRED	REVEL<br>SCORE	MaxEntScan	Global MAF	KRGDB	
*SB422-823	OTOF<br>NM_001287489.2<br>NP_001274418.1	AR	c.2521G>A:p.Glu841Lys<br>chr2:26700042<br>rs772729658	heterozygous	24	0.745	NA	T=0.000029<br>(3/102728, ExAC)	NA	Pathogenic<br>PS4, PM2, PM3, PP1,<br>PP3, PP4
SB390-754	MYO15A<br>NM_016239.3<br>NP_057323.3	AR	c.419delA:p.Lys140Serfs*304<br>chr17:18022533<br>rs750130520	heterozygous	NA	NA	NA	delA=0.000009 (1/111296,<br>ExAC)	NA	Likely Pathogenic<br>PVS1, PM2
			c.9478C>T:p.Leu3160Phe<br>chr17:18064722<br>rs140029076	heterozygous	23	0.729	NA	T=0.006870<br>(829/120676, ExAC)	0.005824	Benign<br>PP3, BA1
SB379-734	LOXHD1<br>NM_144612.6<br>NP_653213.6	AR	c.3748+1G>A<br>chr18:44122689<br>dbSNP ID: NA	heterozygous	33	NA	WT:6.34<br>MT:1.84	NA	NA	VUS<br>PM2, PP3
			c.1270+6_1270+7delTG<br>chr18:44174288_44174289del<br>dbSNP ID: NA	heterozygous	14.59	NA	WT:9.72<br>MT:6.91	NA	NA	VUS<br>PM2, BP4

Supplementary Table S2. Details of subjects with CNS lesions on imaging without confirmation of etiologic diagnosis

IAC MRI abnormality	Clinical diagnosis	Number
**Periventricular leukomalacia**	Ischemic encephalopathy	1
**Periventricular leukomalacia**	Chr.18q21.3 deletion	1
**Periventricular leukomalacia With pachygyria or polymicrogyria**	None	2
**Diffuse brain atrophy**	Meconium aspiration	1
**Cystic cerebromalacia**	Neonatal intracranial hemorrhage	1
**Multifocal variable sized T2 high signal intensity lesions at both cerebral subcortical white matter**	Viral encephalitis or autoimmune disease	1
**Asymmetric size of lateral ventricle with thinning of corpus callosum**	Wolf-Hirschhorn syndrome (Chr. 4p16.3 deletion)	1
**Bilateral symmetrical T2 high signal lesions in both globus pallidus**	None	1
